# Ultrasonographic Investigation of Human Fetus Responses to Maternal Communicative and Non-communicative Stimuli

**DOI:** 10.3389/fpsyg.2016.00354

**Published:** 2016-03-16

**Authors:** Gabriella A. Ferrari, Ylenia Nicolini, Elisa Demuru, Cecilia Tosato, Merhi Hussain, Elena Scesa, Luisa Romei, Maria Boerci, Emanuela Iappini, Guido Dalla Rosa Prati, Elisabetta Palagi, Pier F. Ferrari

**Affiliations:** ^1^Associazione 9 Mesi Ed OltreParma, Italy; ^2^Department of Neuroscience, University of ParmaParma, Italy; ^3^Natural History Museum, University of PisaPisa, Italy; ^4^Centro Diagnostico Europeo, ‘Poliambulatorio Dalla Rosa Prati’Parma, Italy; ^5^Institute of Cognitive Sciences and Technologies, Consiglio Nazionale delle RicercheRome, Italy

**Keywords:** mother communication, voice, matching system, acoustic-visual integration, mouth movements

## Abstract

During pregnancy fetuses are responsive to the external environment, specifically to maternal stimulation. During this period, brain circuits develop to prepare neonates to respond appropriately. The detailed behavioral analysis of fetus’ mouth movements in response to mothers’ speech may reveal important aspects of their sensorimotor and affective skills; however, to date, no studies have investigated this response. Given that newborns at birth are capable of responding with matched behaviors to the social signals emitted by the caregiver, we hypothesize that such precocious responses could emerge in the prenatal period by exploiting infants’ sensitivity to their mother’s voice. By means of a two-dimensional (2D) ultrasonography, we assessed whether fetuses at 25 weeks of gestation, showed a congruent mouthmotor response to maternal acoustic stimulation. Mothers were asked to provide different stimuli, each characterized by a different acoustic output (e.g., chewing, yawning, nursery rhymes, etc.) and we recorded the behavioral responses of 29 fetuses. We found that, when mothers sang the syllable LA in a nursery rhyme, fetuses significantly increased mouth openings. Other stimuli provided by the mother did not produce other significant changes in fetus’ behavior. This finding suggests that fetuses are sensitive only to specific maternal vocalizations (LA) and that fetal matched responses are rudimentary signs of early mirroring behaviors that become functional in the postnatal period. In conclusion, fetuses seem to be predisposed to respond selectively to specific maternal stimuli. We propose that such responses may play a role in the development of behavioral and emotional attunement with their mothers long before birth.

## Introduction

Researchers have been interested in cognitive and social abilities of the fetus since the late 1800s (review by Kisilevsky and Low, 1998). More intense investigations, however, started by the end of the XXth century with the development of fetal physiological monitoring technology and innovations in ultrasound technology, which created new prospects in the study of fetal behavior (Kurjak et al., 2004). During the embryonic stage, the placenta limits the perceptual information necessary for the developing brain and influences the behavior of the newborn and his responses to external stimuli (Smotherman and Robinson, 1988; Lecanuet et al., 1995; Gottlieb, 1997). In fact, as argued by Hepper and Shahidullah (1994), analyses of fetal behaviors in healthy populations reflect that the process of functional development begins in the prenatal period with behaviors emerging and developing continuously over gestation and childhood.

### Fetal Sensitivity to Acoustic Stimuli and Mother’s Vocalizations

In humans, for example, prenatal exposure to the maternal voice or specific speech sequences influences the way vocal and speech sounds are processed by the newborn (DeCasper and Spence, 1986; Fifer and Moon, 1995). Several studies about fetal perceptual skills have shown fetal auditory abilities, including voice discrimination. By analyzing body movements, Hepper et al. (1993) discovered that fetuses were unable to discriminate between their own mother and a stranger’s voice speaking to them through a speaker, but could discriminate between their mother’s voice played through a speaker and the live mother’s voice. DeCasper et al. (1994), measuring fetal heart rate (FHR) changes, found that fetuses could discriminate between a tape-recorded familiar rhyme (recited aloud daily by the mother during the pregnancy) and a novel rhyme (control). Kisilevsky et al. (2003), measuring both FHR and body movements, highlighted that when fetuses were exposed to a tape recording of their mother reading a passage, they showed a 5-bpm increase in heart rate over the first 20 s following voice onset, an increase that was sustained until the end of the recording span. In contrast, they observed a decrease in heart rate of 4-bpm when fetuses were exposed to an unfamiliar women’s voice reading the same story passage. Moreover, further studies have demonstrated that fetal responsivity is highly affected by the physiological state of both mother and fetus. In fact, Voegtline et al. (2013) confirmed that near-term fetuses displayed an orienting response to their mother reading aloud, shown by a reduction in motor activity and a deceleration in FHR response within 30 s after she began reading. These results show that the fetus is able to learn prenatally and suggest that fetuses are sensitive to the communicative input of the mother. They also indicate a possible role for prenatal experience of voices in subsequent language development and attachment.

### Fetuses’ Intentional Motor Skills and Early Social Communication in the Early Postnatal Period

These studies highlight that third-trimester human fetuses are not passive and neutral listeners; rather, they are capable of reacting and retaining information about particular auditory and/or olfactory/gustatory stimuli detectable from the uterine environment and often actively respond to these stimuli (Pedersen and Blass, 1982; Smotherman, 1982; Smotherman and Robinson, 1987; Hepper, 1988; Molina et al., 1995; Mennella and Beauchamp, 1999). Furthermore, several recent kinematic studies demonstrate that human fetuses display an early development of action planning (Patrick et al., 1980; D’Elia et al., 1998, 2001). Effectively, by 22 weeks, fetal movements, traditionally described in terms of reflexes rather than actions, are not uncoordinated or un-patterned, but are directed or aimed at specific targets, suggesting a primitive motor planning process already operating in the fetus during the prenatal period (Zoia et al., 2007). In this regard, Castiello et al. (2010) explored whether the propensity to socially interact is already present before birth. They reported that twin fetuses, in the 14 week of pregnancy, display movements oriented toward their twin with kinematic characteristics different from movements oriented toward the uterine wall or toward their own body; in particular, twins exhibited a higher degree of accuracy in their movements performed toward the eye or mouth regions of their twin sibling than for self-directed movements.

The neonatal period is a unique, crucial time in development (Nagy et al., 2013). During the postnatal period, mothers modify their patterns of behavior when communicating with their neonates, for example by exaggerating and repeating facial expressions (Trevarthen, 1974, 1979; Stern, 1985; Tronick, 1989) or by speaking to infants in special adapted ways defined “baby-talk” or “motherese” (also called infant-directed speech) characterized by short, spaced utterances, peculiar voice timbres, and higher and more modulated voice pitches (Murray et al., 2010). Neonates are highly sensitive to these stimuli and often respond to the caregiver by changing the tone of the muscles, by orienting their gaze toward the mother and by imitating the same facial patterns (Trevarthen and Aitken, 2001; Murray et al., 2010; Simpson et al., 2014). This expressiveness includes various facial expressions of emotion, lip and tongue movements, and active shaping of the mouth, which help adults to understand the nature of their infant’s needs (Trevarthen, 1979). It is still unknown how these competences could emerge in the postnatal period and whether we could track some of these skills prenatally when fetuses are clearly capable of responding to communicative signals.

Neonates’ imitation of facial gestures further demonstrates their capacity to be attuned to the most relevant social signals already soon after birth (see Simpson et al., 2014, for a critical review). Functionally, this represents a remarkable finding, highlighting that the newborn is not a passive recipient, but is actively socially engaging in intersubjective exchanges (Meltzoff and Moore, 1994; Barr et al., 1996). Anecdotal observations and reports from obstetricians, gynecologists and teachers involved in prenatal courses, document mothers communication with their fetuses, principally by talking and singing directly to the baby, or by pampering the ventral region. Do these stimuli elicit any response in the fetus? Do they play any role in organizing brain structures involved in intentional behavior and communication? Intrauterine recordings show that maternal speech and heartbeats are audible in the uterus (Querleu and Renard, 1981; Richards, 1990; Kisilevsky et al., 1993). In sum, these and other reports mentioned in the previous sections, demonstrate that fetuses are very sensitive to mothers’ speech and, through this exposure, can learn several aspects of sounds and vocalizations that will be important in their postnatal life.

### Objectives of the Current Study

As reported above, the mother’s voice influences the general movements of the fetus as well as other physiological parameters. These studies, however, never took into account the possible facialmotor responses of the fetuses as they may reflect possible affective responses. In fact, soon after birth, neonatal facial movements and gestures can occur in response to mother vocalizations (Keller et al., 2008). Such responses probably involve competences regarding infants’ capacity to recognize voices and to learn specific aspects of early social interactions, which very likely develop in the prenatal period.

Based on these reports, the aim of our study was to verify, by means of a two-dimensional (2D) ultrasonography, whether, during the prenatal period, the fetus is sensitive to maternal acoustic stimulations. Unlike previous studies, we conducted a behavioral analysis focused on fetal facial movements in response to specific acoustic stimuli. Therefore, one of the goals of our study was to assess if fetuses specifically respond to communicative and non-communicative stimuli. Among other stimuli, we assessed the effects of specific vocalizations sung by the mother in a nursery rhyme and with two different syllables as basic vocal components. From our previous unpublished observations we noticed that nursery rhymes often elicited stronger behavioral responses in fetuses than other types of stimuli, such as the reading of a book, or chewing. Mothers commonly use nursery rhymes during pregnancy but, to our knowledge, there are no systematic studies investigating this phenomenon. Mothers were instructed to emit different vocalizations or to produce sounds with the mouth through chewing activities. Vocalizations were nursery rhymes (sung in syllables) and single syllables (LA or LU) that were repeated several times by the mother. The use of syllables with specific vowels has advantages in allowing control over the linguistic content, and also reducing the acoustic stimuli to very basic phonemes, which can be easily distinguished from other types of acoustic stimuli.

In this study we hypothesize that the sensorimotor mechanisms involved in the auditory and motor processing are somehow coordinated, and contribute to the fetuses’ responses to maternal vocalizations. Even though visuomotor skills start to form throughout the course of prenatal life (Del Giudice, 2011), the visual system develops relatively late in gestation compared with the tactile, auditory and olfactory systems. Hence, our research specifically focused on the acoustic channel, since this sensory modality, as previously observed, seems to play an important role in helping the fetus to orient to and efficiently respond to external stimuli.

## Materials and Methods

### Ethic Statement

Experimental procedures were approved by the Institutional Review Board at the University of Parma and the local Ethical Committee, and were in accordance with the Declaration of Helsinki (Seventh revision, October 2013). Participants were fully informed about the aims and procedures of the research and gave written consent to participate in the study.

### Experimental Setting

**FIGURE 1:**
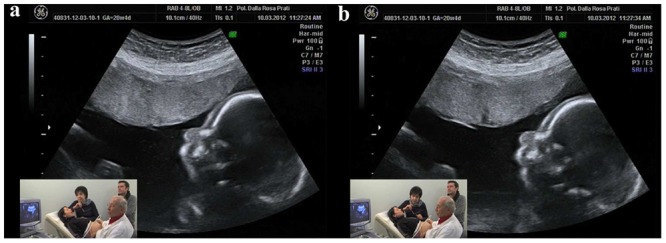
**An ultrasonographic image and the experimental setting.** The small frame shows the mother and ecographer, together with the operator (AFG) and the father. **(a)** Shows the mother while opening the mouth and **(b)** shows the fetus’ congruent response of mother’s mouth opening.

Experiments were carried out at two centers—*Centro Diagnostico Europeo Dalla Rosa Prati* (Parma, Italy) and *Studio Boerci* (CesanoBoscone, Italy)—and involved 29 expectant mothers who were attending the *Associazione Nove Mesi Ed Oltre* (Parma, Italy), an organization fostering prenatal education to expectant parents. Before entering the ultrasound room, mothers completed a questionnaire aimed at gathering information on previous childbirths, unsuccessful pregnancies, and other general biographical data. All 29 women (mean age = 32 years old, ±0.96 SEM) were healthy and had normally evolving pregnancies, with 19 of them being *primiparae*. None of the mothers was under pharmacological therapy at the time of the experiment or had been previously hospitalized for psychiatric disorders. Ultrasound tests were performed between 19 and 27 weeks of pregnancy (mean gestational phase = 23.58 weeks ±0.40 SEM) with the women lying on their backs in a semi-recumbent position. Experiments were conducted in the early afternoon in a quiet room and in the presence of the father, two cameramen, the ultrasonographer and an operator (see **Figures 1a,b** for the ultrasonographic image and the experimental setting). Ultrasound tests were performed by means of the 2D ultrasonograph Voluson 730 PRO BT 08. Following the experimental time schedule, the operator instructed the mother as to which stimulus to produce and its duration. Each test lasted 30 min. Ultrasound and mothers’ videos were then joined in a single video and synchronized to temporally link fetal behaviors and maternal stimuli.

### Video-Analysis and Data Collection

The video-analysis was conducted under blind conditions. That is, the ultrasound and the parents’ videos were analyzed separately to avoid any possible observational bias.

The ultrasound videos were analyzed frame-by-frame by three experimenters who underwent a training period to reach an acceptable level of observational reliability. During the training period, three observers independently analyzed the same videos. Each observer coded the ethogram items and the exact second in which the behavior occurred. Then the records were compared and Cohen’s *k* values were calculated for each behavior. Kappa coefficients were computed to assess the agreement for each behavioral category, and all Cohen’s *k* were >0.7.

### Operational Definitions and Statistics

During the experiment, mothers were invited to perform mouth movements and to emit particular vocalizations (see **Table 1** for the list of stimuli and their descriptions). Stimuli were randomly presented.

**Table 1:** List and definitions of both maternal stimuli and fetal behaviors.

	Initials	Description
**Maternal stimuli**		
La	LA	The mother sang the LA syllable in a nursery rhyme 10 times in 10 s (= 1 bout). The mother performed 3 bouts. Each bout was separated by a pause of 10 s.
Lu	LU	The mother sang the LU syllable in a nursery rhyme 10 times in 10 s (= 1 bout). The mother performed 3 bouts. Each bout was separated by a pause of 10 s.
Mother open mouth	MOM	The mother opened her mouth 10 times in 10 s without emitting any sound (= 1 bout). The mother performed 3 bouts. Each bout was separated by a pause of 10 s.
Mother chew	MCH	The mother chewed a wafer or a biscuit for 1 min (= 1 bout). The mother performed 2 bouts. Each bout was separated by a pause of 30 s.
Simulated yawn	SYW	The mother simulated 3 yawns in 1 min.
**Fetal Behavior**		
Fetus open mouth	FOM	The baby widely opened the mouth and closed it immediately after having reached the maximum opening.
Fetus chew	FCH	The baby made chewing movements. The mouth was narrowly opened and the lips were slightly pressed in a repeated way.
Neck extension	NEX	The baby extended the neck upward by distancing the chin from the chest.
Lip protrusion	LP	The baby pushed out the lips while maintaining the mouth closed.
Yawning	YW	The baby yawned. Yawning was characterized by the following sequence of actions: (1) a slow opening of the mouth; (2) a long-lasting period of maximum opening (at least 4 s) and (3) a slow closing of the mouth. Yawning was usually accompanied by neck extension.

**FIGURE 2:**
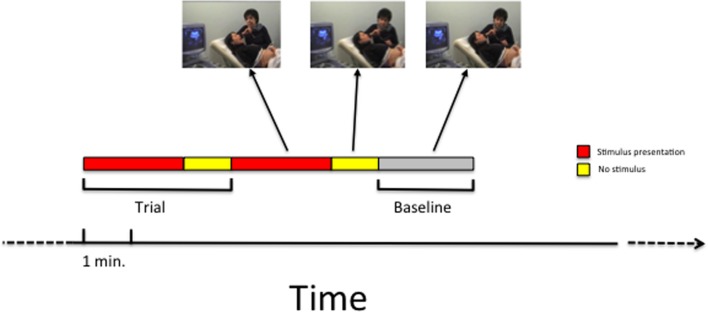
**A schematic illustration of the time sequence of trials and baseline that was employed in the study**.

Each experimental trial lasted the duration of the stimulus presentation (see **Table 1**) plus 1 min following the end of the stimulus presentation. This extra minute was considered part of the trial in order to monitor fetal responses also in the period following the stimulus presentation. Two subsequent experimental trials were each separated by 2-min baseline blocks (baseline condition), defined as periods in which no stimulus was presented. During the baseline condition, the mother made no mouth movements or vocalizations. A schematic illustration of the paradigm is presented in **Figure 2**.

A standard longitudinal section view of the fetus was obtained so that the head and the upper body were entirely visible. During the ultrasound video analysis, the experimenter recorded each behavior performed by the fetus (see **Table 1** for the list of fetal behaviors and their descriptions), including the exact second in which the behavior occurred, its duration and frequency (number of behaviors/seconds of observation). These behaviors were subsequently analyzed in relation to the mother’s stimulation.

We recorded all the fetal behaviors listed in **Table 1** performed during the stimulus presentation and in the minute following the end of that stimulus (experimental trial). Two response events were counted as different events when they were separated by at least 3 s. See **Table 1** for the duration of each experimental trial.

When data did not violate assumptions of normality, we applied parametric statistics. In all other analyses, we carried out non-parametric tests.

The following comparisons have been employed for the statistical analyses.

#### General Activity Test: Experimental vs. Baseline Condition

A paired *t*-test compared fetal activity between experimental and baseline conditions (*k* = 2). The two conditions are defined as follows: (1) LA + LU + MOM + MCH + SYW: Experimental trial (all the behaviors performed by the mother along with the total duration of the test were used as stimuli condition); (2) Baseline condition (the mother made neither any mouth movement or emitted any kind of vocalization).

Friedman’s two-way analysis of variance was used to test for the congruence of fetal response to LA and MCH stimuli across three different conditions.

In the following tests, three conditions were compared in order to test whether the fetus mouth behavior was congruent with that of the mother.

#### Congruence Test: LA vs. MOM vs. ALL OTHER

LA: experimental trial (the mother sings the LA syllable in a nursery rhyme); MOM: first control condition(the mother opens the mouth without emitting any sound, with the mouth opening employing the same motor pattern as in LA experimental trial); ALL OTHER: second control condition (LU + MCH + SYW; see **Table 1** for the definitions).

#### Congruence Test: MCH vs. MOM vs. ALL OTHER

MCH: experimental trial (the mother chews a piece of food – wafer or a biscuit); MOM: first control condition (the mother opens the mouth without emitting any sound, the opening the mouth has the same, or similar, motor pattern as the MCH experimental trial); ALL OTHER: second control condition (LA + LU + SYW; see **Table 1** for the definitions).

In case of significance across the three conditions, we ran the Dunnett’s multiple comparison test (*post hoc* test) to determine which pairs of conditions differed significantly.

## Results

The sample size used for the different statistical analyses changed because we had to exclude some fetuses when they were not clearly visible (e.g., the posture or excessive movements of the fetus did not allow recording good quality images of the face) or when the fetus was sleeping during stimulus presentation. We also discarded instances in which the mechanical maneuver from the doctor with the probe could have elicited behavioral responses to the fetus. For these reasons the discard rate has been particularly high. We discarded 16 subjects in the congruence test (LA vs. MOM and ALL). We discarded 19 subjects in the other congruence test (MCH vs. MOM and ALL). The high discard rate was somehow unexpected. This was also partly due to our conservative criteria for inclusion of valid trials and also to scarce information from previous literature assessing behavioral responses of fetuses at this gestational age.

**FIGURE 3:**
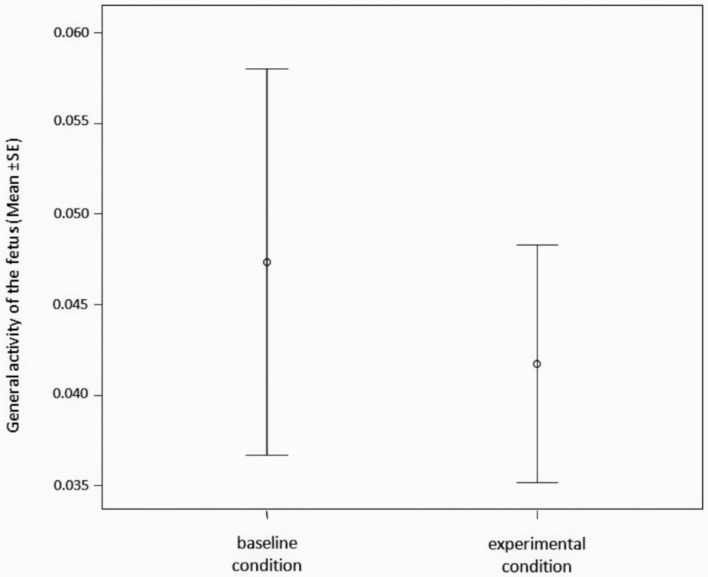
**Time frequency of the general behavioral activity of the fetus (number of FOM + FCH + NEX + LP + YW/seconds of observation) during experimental (maternal stimuli administration: LA + LU + MOM + MCH + SYW) and during baseline condition (absence of any kind of stimulus)**.

The general activity of the fetus (time frequency of FOM + FCH + NEX + LP + YW – see **Table 1** for descriptions) did not differ between the baseline (absence of any kind of stimulus) and the experimental conditions (presence of maternal stimuli administration: LA + LU + MOM + MCH + SYW – **Table 1**) (Paired *t*-test; *t* = 1.025; df = 25; *p* = 0.315) (**Figure 3**).

**FIGURE 4:**
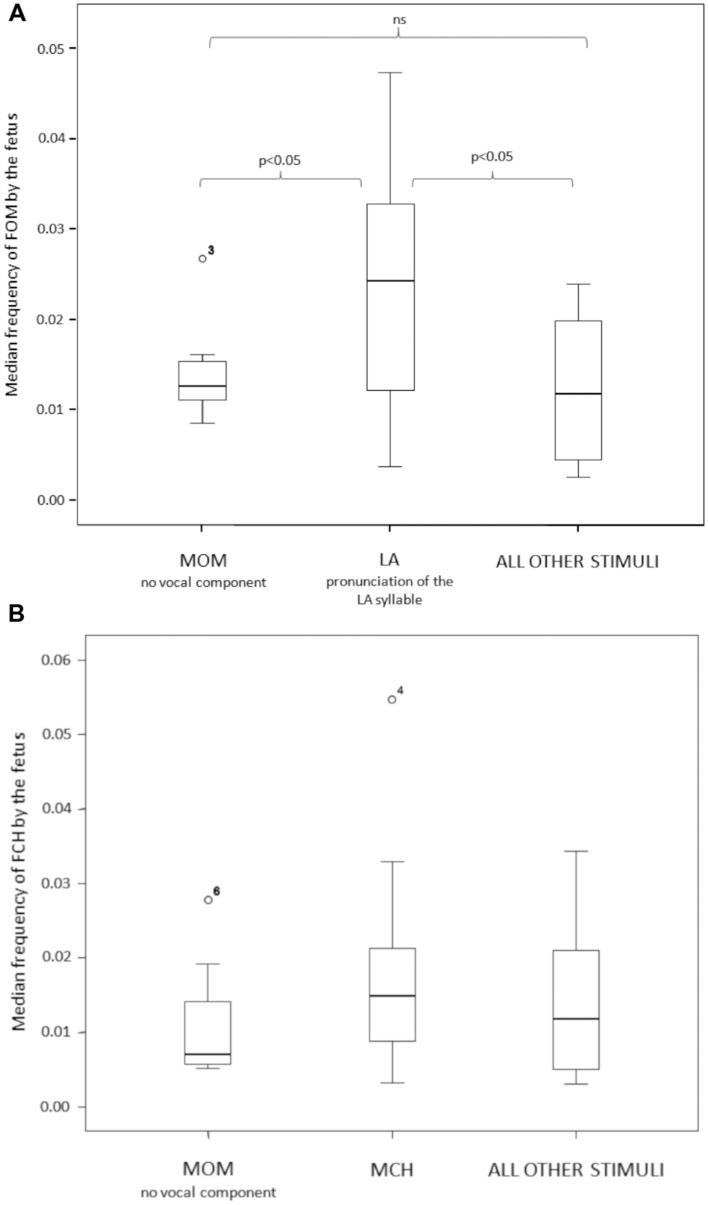
**(A)** The open mouth (FOM) response of the fetus differed across the three stimulus conditions (LA, MOM, ALL OTHER). The fetus opened the mouth (FOM) more frequently when the mother sang the LA syllable in a nursery rhyme. **(B)** The chew response of the fetus (FCH) did not significantly differ as a function of the maternal stimuli proposed (MCH vs. MOM vs. ALL OTHER).

To test for the congruence of the fetus response to the LA maternal stimulus we compared the open mouth response of the fetus (FOM) across three maternal stimulus conditions: LA, MOM and ALL OTHER (LU + MCH + SYW). MOM and ALL OTHER represent a double control for the LA experimental condition. The response differed as a function of the stimulus presented (Friedman test χ^2^ = 6.00; *n* = 13; df = 2; *p* = 0.05; Effect size via Kruskal–Wallis Test: MOM vs. LA = 0.610; LA vs. ALL OTHER = 0.649; MOM vs. ALL OTHER = 0.087) (**Figure 4A**). The Dunnett’s test revealed that FOM response to the LA stimulus was statistically higher than the response to MOM and to ALL OTHER stimuli conditions (LA vs. MOM: *q* = 2.49, *p* < 0.05; LA vs. ALL OTHER: *q* = 2.35, *p* < 0.05). The FOM response to MOM and ALL OTHER conditions did not differ (*q* = 0.83, *p* > 0.05).

We also tested for the congruence of the fetus chewing response (FCH) to maternal chewing stimulus (MCH). For this purpose, we considered the following conditions: MCH, MOM and ALL OTHER stimuli pooled together (LA + LU + SYW). MOM and ALL OTHER represent a double control for the MCH experimental condition. The FCH response did not differ as a function of any of the stimuli proposed (Friedman test χ^2^ = 2.40; *n* = 10; df = 2; *p* = 0.368; Effect size via Kruskal–Wallis Test: MOM vs. MCH = 0.435; MCH vs. ALL OTHER = 0.370; MOM vs. ALL OTHER = 0.274) (**Figure 4B**).

## Discussion

Our results showed that the fetus is responsive to maternal vocal stimulation during pregnancy. However, the overall activity of the fetus was similar between the baseline and the stimulus period, thus suggesting that in both periods fetuses were vigilant and active. One of the most interesting findings is that the fetus is particularly responsive to one acoustic stimulus. In particular, when the mother emits the sounds LA (sung in a nursery rhyme), the fetus responds with a greater frequency of mouth openings (FOM) compared to the MOM and ALL OTHER stimuli. In ALL OTHER conditions, the syllable LU sung in a nursery rhyme was also included, however, the sound characterized by the syllable LU did not trigger any behavioral response. The syllables LU and LA only differed for the vocal component because the lullaby was the same.

It is unclear, however, whether the effect we found is a simple response to emotional arousal or a reflex-like response. A possible way to test this hypothesis might be to analyze FHR variations in response to the mother or to other individuals (father or strangers) speaking and communicating with the fetus. A differential heart rate response to the mother vs. a stranger’s (or less familiar) voice could provide evidence that these behavioral outcomes are accompanied by emotional arousal involving the autonomic system. Our findings thus add important information to previous investigations that reported fetuses’ responses to maternal vocalizations (Kisilevsky et al., 2009; Voegtline et al., 2013). These studies, however, assessed fetuses’ heart rate or general movement, showing that the responses could be interpreted as orienting behaviors, dependent on fetus or mother’s state during the baseline (Voegtline et al., 2013). Our analysis instead were more focused on the infant’s mouth movements and took into account the resting state of the mother and the level of vigilance of the fetus. A recent study also found that infants are sensitive to mother’s voice and they change their behavior accordingly (Marx and Nagy, 2015). However, no changes in mouth movements were recorded while the mother read a story.

Looking at the parents’ behavioral responses during testing we noticed large inter-individual variability: some mothers and fathers showed a lot of emotional involvement during the session, unlike other parents who seemed a bit inhibited or not at ease, perhaps because of the presence of foreign operators. Therefore, it is possible that mothers’ stress levels may have influenced fetal responses. However, this aspect would require further analysis with a larger sample of mothers as well as the inclusion of the participation/empathy measurements as a variable.

One intriguing question is how to interpret the finding of fetal matching responses. One possibility, consistent with our original hypothesis, is that fetuses at this gestational age might have already developed some rudimentary forms of motor resonance, which involves the capacity to activate motor representation similar to that of the model (i.e., the mother). The presence of a mirror mechanism has been reported first in monkeys and then in humans (Rizzolatti and Craighero, 2004) and involves the cortical structures of the motor/premotor and parietal cortices. The capacity to match perceived and executed behaviors is a very precocious ability in humans and other primates. For example, the presence of early facial imitative behavior at birth has been reported for both humans and monkeys (Meltzoff and Moore, 1977; Ferrari et al., 2006) and it may be supported by a mirror mechanism as recently described by means of electroencephalogram in newborn monkeys (Ferrari et al., 2012). Interestingly, several studies have reported contagious crying in human newborns (Dondi et al., 1999; Geangu et al., 2010). These studies suggest that, at birth, different sounds of crying are efficient in eliciting contagious crying reactions in newborns (Simner, 1971), and those cries which more closely resemble the characteristics of the listener’s age seem to elicit more affect sharing, more facial and vocal distress (Simner, 1971 and Martin and Clark, 1982). Although these studies found a matching mechanism coupling visual and motor information, it is also possible that, given the early development of the acoustic system in fetuses, such matching could also be present very early in the prenatal and postnatal period, involving other sensory modalities. It is also possible that these early responses could rely on subcortical mechanisms since the corticogenesis is still not completed at 25 weeks of gestation (Kostovic et al., 2002). Thus, more studies are necessary to better understand the possible relation between the described behavioral phenomena, the cortical motor development and the activity of other subcortical structures involved in the processing of biologically meaningful information.

It is even more intriguing to understand why fetuses display such responses. They have no apparent function, at least in the communicative domain. We might speculate that these responses are rudimentary signs of early motor resonance behaviors that could become functional in the postnatal period, helping the infant to establish behavioral and emotional attunement with the mother, and facilitating the newborn to respond more selectively to the stimuli of the mother. Such early imitative responses might facilitate positive social affect between the mother and the infant (Ferrari et al., 2006; Simpson et al., 2014). Moreover, infants can distinguish between the mother’s and a stranger’s voice, based on their prenatal acoustic experience. This learning process therefore starts during the prenatal period. The fact that fetuses responded only to LA, and not to LU, could be due to the fact that the former are more familiar to infants because the mother uses this syllable in nursery rhymes more often than LU. However, we do not have data available to verify this hypothesis. Other alternative explanations, but not necessarily in contrast, could be related to intrinsic acoustic features of LA sounds compared to LU, with the former that could be more easily perceived by fetuses in uterus due to intrinsic features of the acoustic stimulus and thus leading to an increase in the arousal state, with the result of an increase mouthing activity. However, this hypothesis needs to be experimentally tested. Interestingly, animal studies have shown that sounds with different characteristics can be differently transmitted in the uterus (Abrams et al., 1995, 1997).

Based on our and others’ findings (Kisilevsky et al., 2009) we hypothesize that, during the prenatal period, the exposure to the sounds and song of a mother might stimulate infants’ capacity to learn and distinguish the mother’s voice and thus facilitate, in the postnatal period, the process of face discrimination based on acoustic and visual stimuli. Responding through mouth opening to mothers’ voice during pregnancy seems to reflect a process of motor resonance, rather than arousal, that could subsequently functionally be exploited in the postnatal period in order to respond more selectively to the mother’s voice and gesture and to promote her social affiliation and positive affect.

Interestingly, a study on infants as young as 12 weeks showed that they were able to imitate specific vocalizations. In this study, infants who were watching videosin which an adult was pronouncing one of three vowels (/a/, /i/ or /u/), responded with vocalizations that perceptually matched those that were presented (Kuhl and Meltzoff, 1996). This clearly shows that infants discriminate acoustically different sounds and that they are also capable of making acoustic-motor transformations in communicative settings. In another study, using a preferential viewing procedure, Kuhl and Meltzoff (1982) showed that 18–20-week-old infants spent significantly more time watching the utterances /i/ and /a/ when they were accompanied by the matching sound. However, the effect was no longer present when vocalizations were replaced by pure tones that were similar in timing and tonality. Together, these findings provide evidence that it is important for infants to learn to associate the face and gesture of an individual with his/her vocalizations. Early experience with mother’s sound during the prenatal period might therefore help infants in the postnatal period in building such audio-visual correspondence while engaged with their mother through face-to-face interactions (see Kuhl and Meltzoff, 1982).

The idea that some behaviors present in uterus with no apparent function could prepare a fetus for postnatal life is not new. Fetuses, for example, show several facial expressions and these expressions undergo maturation during gestation (Craig et al., 1994; Reissland et al., 2011). A recent study found that fetuses at around 24 weeks of gestation display facial expressions of emotions related to pain and/or distress (Reissland et al., 2013). These authors proposed that, even though these facial expressions have no communicative meanings, they become adaptive in the postnatal period as they could alert the caregiver about negative experiences of the newborn.

Our findings are also consistent with a possible relation and continuity between fetal mouth cyclic movements, babbling and early speech forms (Vihman et al., 1985, 1986). Babbling, the random production of consonant-like sounds in babies generally associated with the vowel A, is a direct result of production of syllabic “frames” by means of rhythmic mandibular oscillation; it first occurs around 6 months of age (Oller, 1980; Stark, 1980; Holmgren et al., 1986; Koopmans-van Beinum and Van der Steldt, 1986) and can be characterized as phonation accompanied by an alternation of closed and open phases of the mouth within spatio-temporal timing patterns appropriate for adult utterance strings. Our results are consistent with the hypothesis that the fetus tends to display a more frequent response to the vowel ‘A’ since babbling represents a crucial first phase of development in word-production ability, and enables us to postulate that a prenatal attentive behavior toward such stimulus types could enhance fetal/infant subsequent phonetic abilities.

Despite these data described an interesting phenomenon in fetuses, we are aware of some limitations of the study. One important aspect that we should consider is the baseline period that probably has been too short and did not allow controlling for possible behavioral effects in several fetuses. This problem has led to discard some videos from the analysis, because the fetuses, after the stimulus presentation, did not return to a baseline activity, probably due to arousal or delayed responses to the stimulation provided by the mother. In addition to this, we also did not control for mother’s state at the time of assessment. Some mothers appeared to be very relaxed and not concerned about the persons involved in the research that were present in the room for the assessment. Others, however, appeared apprehensive. This inter-individual variability might have had an impact on mothers’ stress responses and the consequent fetuses responses or inhibited behaviors. Lastly, although we found fetus reactivity in several cases, we also recorded fetal reduced responsiveness and resting behaviors in many of them. Such reduced general activity likely reflects cycle of rest-activities, which led to a high discard rate. Future studies should therefore take into account such limitations in carrying out behavioral research in fetuses.

## Author Contributions

All authors listed, have made substantial, direct and intellectual contribution to the work, and approved it for publication.

## Conflict of Interest Statement

The authors declare that the research was conducted in the absence of any commercial or financial relationships that could be construed as a potential conflict of interest.
